# Artefenomel Regioisomer RLA-3107 Is a Promising Lead for the Discovery of Next-Generation Endoperoxide Antimalarials

**DOI:** 10.1021/acsmedchemlett.3c00039

**Published:** 2023-04-04

**Authors:** Brian R. Blank, Jiri Gut, Philip J. Rosenthal, Adam R. Renslo

**Affiliations:** † Department of Pharmaceutical Chemistry, University of California, San Francisco, 600 16th Street, San Francisco, California 94158, United States; § Department of Medicine, San Francisco General Hospital, University of California, San Francisco, San Francisco, California 94143, United States

**Keywords:** Antimalarials, artefenomel, trioxolanes, lead optimization, stereoselective synthesis

## Abstract

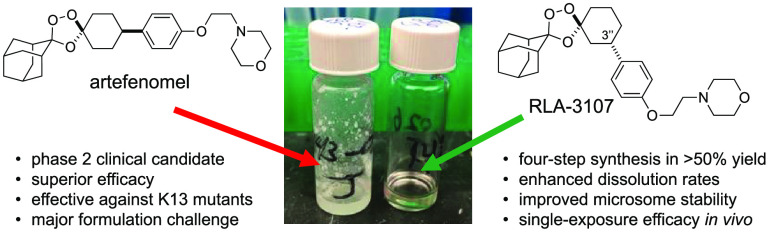

Clinical development of the antimalarial artefenomel was recently halted due to formulation challenges stemming from the drug’s lipophilicity and low aqueous solubility. The symmetry of organic molecules is known to influence crystal packing energies and by extension solubility and dissolution rates. Here we evaluate RLA-3107, a desymmetrized, regioisomeric form of artefenomel *in vitro* and *in vivo*, finding that the regioisomer retains potent antiplasmodial activity while offering improved human microsome stability and aqueous solubility as compared to artefenomel. We also report in vivo efficacy data for artefenomel and its regioisomer across 12 different dosing regimens.

Malaria remains a cause of significant morbidity and mortality in sub-Saharan Africa and Southeast Asia.^1^ Due to increasing resistance of *Plasmodium falciparum* to available drugs, new antimalarial agents are urgently needed. The synthetic 1,2,4-trioxolane artefenomel^2^ (**1**, Figure 1) was until very recently a leading candidate to replace the sesquiterpene artemisinin and its semisynthetic analogs in artemisinin combination therapy (ACT) for uncomplicated malaria. The prolonged exposure profile of artefenomel in malaria patients, combined with modeling in ring-stage survival assays,^3−5^ predicted for clinical efficacy of artefenomel against artemisinin partial resistant parasites bearing mutations in PfKelch13 (K13).^6,7^ However, challenges^8^ in formulating the necessary 800 mg human dose for oral administration ultimately led to termination of clinical evaluation of artefenomel. A second candidate, OZ609,^4^ has also been withdrawn from clinical development for undisclosed reasons. Thus, currently the Medicines for Malaria Venture (MMV) pipeline is devoid of novel endoperoxide-class drug candidates, despite the unquestioned success of ACT, and over two-decades of sustained efforts by a number of groups^9^ to identify improved, synthetic endoperoxide drug candidates.

**Figure 1:**
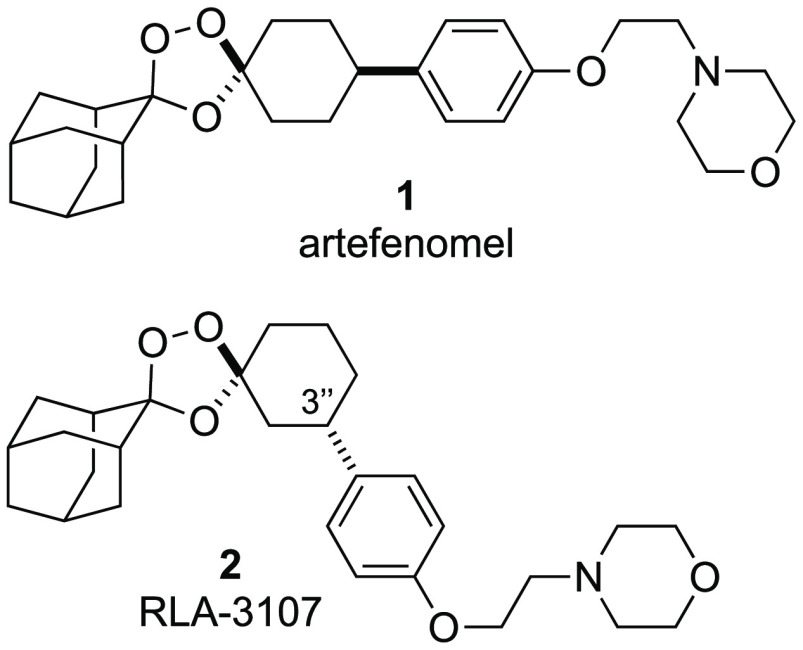
Structures of the clinical-stage antimalarial artefenomel (**1**) and its 3″-regioisomer RLA-3107 (**2**) described herein.

With the goal of identifying endoperoxides with improved drug-like properties, our laboratory has synthesized and reported^10,11^ on the pharmacokinetic and pharmacodynamic properties of desymmetrized congeners of arterolane, the only 1,2,4-trioxolane to have received regulatory approval, albeit only in India. In this earlier work, we identified analogs with improved oral efficacy compared to arterolane, including examples that afforded single-exposure cures at a dose of 50 mg/kg in the *Plasmodium berghei* mouse model.^11^ More recently, O’Neill and co-workers described desymmetrized analogs of the tetraoxane antimalarials E209 and E205, which bear an artefenomel-like side chain. The desymmetrized analogs in general exhibited markedly lower melting points than the symmetrical progenitors, while an analog of E205 exhibited significantly improved solubility in simulated gastric fluid and an improved pharmacokinetic profile.^12^

Here we describe an efficient (4 steps, 50% overall yield) synthesis of RLA-3107 (**2**), a desymmetrized regioisomer of artefenomel. We compared **1** and **2** across *in vitro* ADME and antiplasmodial assays, and in the *P. berghei* mouse malaria model using 12 different oral dosing regimens. Notably, we found that the human microsome stability, aqueous solubility, and dissolution properties of **2** were markedly improved over **1**, while **1** was still superior in terms of oral efficacy. Overall, these studies reveal an artefenomel-adjacent pharmacophore with the potential to address the physiochemical liabilities that derailed artefenomel, an otherwise very promising antimalarial drug candidate.

The unusual pharmacology of 1,2,4-trioxane and 1,2,4-trioxolane antimalarials is contingent on an initial Fenton-type reaction of the endoperoxide function with parasite sources of ferrous iron, likely including ferrous heme iron.^13,14^ Rates of ferrous iron reactivity are in turn dictated by conformational mobility of the cyclohexane ring in either shielding (conformer I) or exposing (conformer II) the endoperoxide function for reaction (Figure 2). Accordingly, *cis*-R^4^ or *trans*-R^3^ side chains have been found empirically to provide a pharmacologically optimal balance of iron stability and reactivity under physiological conditions.^10,11,15^

**Figure 2:**
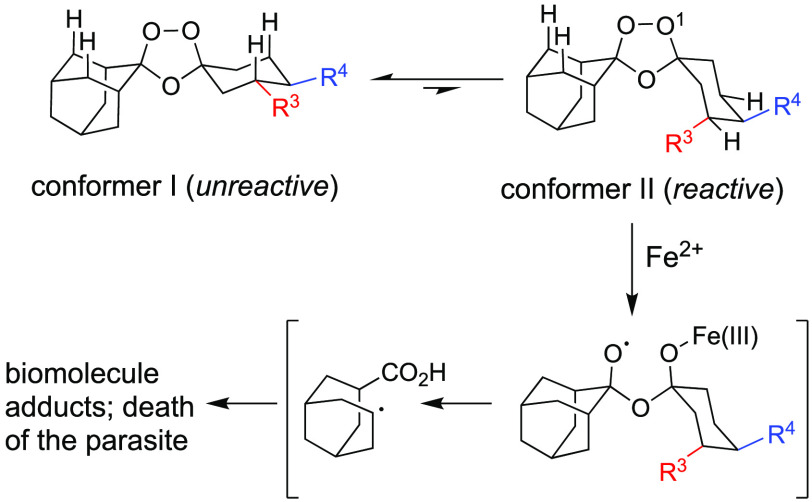
Conformational effects on iron reactivity and antimalarial pharmacology of 1,2,4-trioxolanes. Arterolane and artefenomel (**1**) possess *cis*-R^4^ side chains and are symmetric about a plane encompassing the trioxolane ring. Desymmetrized artefenomel regioisomer RLA-3107 (**2**) described herein bears *trans*-R^3^ substitution.

The Griesbaum co-ozonolysis reaction commonly used to prepare 1,2,4-trioxolanes is an intrinsically diastereoselective process (9:1 d.r. is typical) affording the desired *cis*-R^4^ or *trans*-R^3^ diastereomers in reactions of C4- or C3-substituted cyclohexanones, respectively.^16^ Full stereocontrol can thus be achieved by employing enantiomerically pure cyclohexanone substrates, as demonstrated in our enantioselective synthesis of arterolane-like *trans*-R^3^ amide^10^ and carbamate^11^ analogs, and in O’Neill’s synthesis of desymmetrized tetraoxanes.^12^

Here, we employed racemic cyclohexanone **3** to ultimately afford (±)-**2** as the desired *trans* diastereomer (Scheme 1). Thus, a dicationic palladium(II) complex obtained by mixing Pd(acac)_2_, dppben, and Cu(BF_4_)_2_·H_2_O successfully catalyzed the 1,4-addition of 4-(acetoxy)phenyl boronic acid to 2-cyclohexen-1-one, affording ketone **3** in 91% yield. Next, Griesbaum co-ozonolysis of **3** with adamantan-2-one *O*-methyl oxime in the presence of ozone at 0 °C afforded 3′′-aryl substituted 1,2,4-trioxolane intermediate **4** in nearly quantitative yield, and a diastereomeric ratio of 8:1. Hydrolysis of the acetate protecting group was achieved in 95% yield by heating at 50 °C with KOH in methanolic THF solvent. Alkylation of phenolic intermediate **5** with 4-(2-chloroethyl)morpholine hydrochloride in the presence of powdered NaOH and (Bu)_4_NHSO_4_ at 55 °C in acetonitrile then yielded (±)-**2** in 62% yield (50% overall yield for four steps). Purification of intermediate **5** and final analog **2** by column chromatography served to remove the last remnants of the minor diastereomer, such that (±)-**2** was obtained as pure *trans* diastereomer, within the limits of ^1^H NMR detection. Since ketone **3** has been prepared previously in enantiopure form,^12^ the synthesis of (±)-**2** described herein is fully amenable to the preparation of enantiomerically pure **2**.

**Scheme 1:**
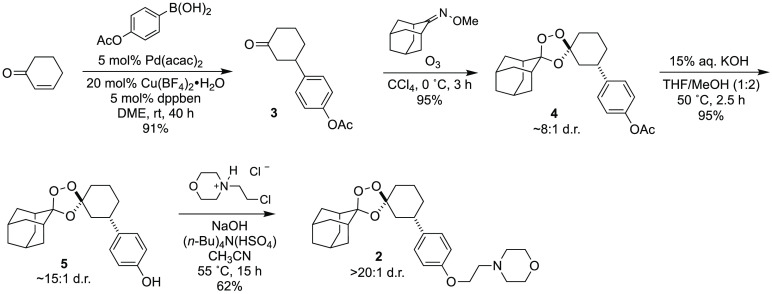
Stereocontrolled Synthesis of Artefenomel Regioisomer *trans*-(±)-**2**

The impact of desymmetrization on the physiochemical properties of **2** was immediately apparent when **1** and **2** were prepared as DMSO stock solutions for *in vitro* testing. Thus, compound **2** was isolated as a clear oil that readily dissolved in DMSO, while **1**, a solid, required heating and sonication to produce a homogeneous DMSO stock solution (Figure 3). Next, **1** and **2** were evaluated in triplicate for antiplasmodial activity in human erythrocyte cultures with chloroquine resistant W2 strain *P. falciparum*, using artemisinin and chloroquine as controls (Table 1).

**Figure 3:**
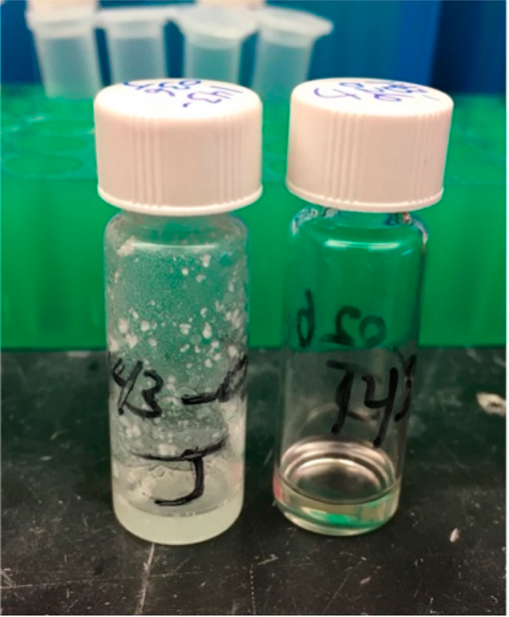
Image illustrating the attempted dissolution of molar equivalents of **1** (left) and **2** (right) in DMSO upon preparing a stock solution for antiplasmodial testing. Compound **1** required heating and sonication to produce a homogeneous solution, whereas **2** dissolved immediately at room temperature.

**Table 1:** *In Vitro* Antiplasmodial Activity and *In Vitro* ADME Properties of **1** and **2**

compounds	W2 *P. falc*. EC_50_^a^	HLM CL_int_ (μL/min/mg)	solubility in PBS pH 7.4 (μM)
1	11.2 ± 0.54 nM	63.7^b^	<2.5^b^
2	24.1 ± 0.62 nM	21.3^c^	24.8^c^
artemisinin	17.3 ± 2.1 nM		
chloroquine	112 ± 7 nM		
verapamil		147.6;^b^ 172.9^c^	
amiodarone			<3^c^
diclofenac sodium			198.8^b^

a Average of three determinations ± standard error in mean.

b Data reported for **1** was generated for the authors by MMV.

c Data generated for this study by Quintara Biosciences, South San Francisco, CA. Verapamil is a positive control in the human liver microsome (HLM) assay. Amiodarone and diclofenac sodium are controls in the solubility assays.

The low-nM EC_50_ values demonstrated by **1** and **2** bracketed the value for the artemisinin control, while chloroquine (CQ) was significantly less potent, as expected for this CQ-resistant parasite strain. Thus, desymmetrized artefenomel regioisomer **2** had similar intrinsic antiplasmodial activity against cultured malaria parasites. This result was consistent with the findings of prior studies of desymmetrized analogs of arterolane and E205/E209 noted above, and with current understanding of trioxolane pharmacology.

Next, we evaluated the human microsome stability and aqueous solubility of **2** and compared the results with values reported^17^ for **1** or provided to us by MMV. We found that **2** exhibited significantly improved solubility in pH 7.4 PBS, as well as reduced clearance in human liver microsomes as compared to **1** (Table 1). These encouraging findings suggested that 3″-aryl substitution offers the potential to improve metabolic and drug-like properties in artefenomel-like antimalarials. The enhanced solubility and qualitatively improved dissolution rates (Figure 3) observed for **2** are particularly significant given that formulation of oral drug substance was the major practical hurdle derailing the clinical development of artefenomel.

Given that the *in vitro* antiplasmodial and ADME data for **2** suggested good potential for *in vivo* activity, we next evaluated the efficacy of **2** in the *P. berghei* mouse malaria model. We used parasitemia-free survival at day 11 (“survivors”) and day 30 (“cures”) as primary and secondary study end points. We explored a range of dosing schedules including repeated dosing (QD × 4 days) at lower administered dose (2–10 mg/kg) and single-dose administration at higher doses (20–80 mg/kg). Drug administration was by oral gavage, with **1** as control in all study arms. The results of selected arms are provided below (Table 2) while the results of all 12 study arms are provided as Supporting Information. When studied with QD × 4 day dosing, **1** produced cures of all animals (5/5) at either the 10 mg/kg or 6 mg/kg doses. The compound was somewhat less efficacious at 4 × 4 mg/kg (4/5 cures), and least so at 4 × 2 mg/kg (1/5 cures). The consistent dose–efficacy relationship of the artefenomel comparator provided high confidence in the results of these studies. Compared to **1**, its regioisomer **2** was notably less efficacious in the QD × 4 day study arm. Thus, at the highest 4 × 10 mg/kg dose, **2** produced 4/5 survivors at day 11, but no cures by day 30, and was ineffective at the lower doses. In the single-dose arms, both **1** and **2** afforded 5/5 cures with a single oral dose of 80 mg/kg. At 40 mg/kg, **1** afforded 5/5 cures while **2** produced 3/5 survivors at day 11 but only one cure at day 30. Notably, **1** produced 5/5 cures with single doses of 30 mg/kg or 20 mg/kg, exhibiting the remarkable *in vivo* efficacy that led to its original selection for clinical development.

**Table 2:** *In Vivo* Efficacy of **1** and **2** in the *P. berghei* Mouse Malaria Model with Oral Dosing at Different Frequencies and Doses^a^

days × PO dose	compound	survivors (day 11)	cures (day 30)
1 d × 80 mg/kg	**1**	5/5	5/5
	**2**	5/5	5/5
1 d × 40 mg/kg	**1**	5/5	5/5
	**2**	3/5	1/5
1 d × 30 mg/kg	**1**	5/5	5/5
	**2**	5/5	0/5
1 d × 20 mg/kg	**1**	5/5	5/5
	**2**	3/5	0/5
4 d × 10 mg/kg	**1**	5/5	5/5
	**2**	4/5	0/5
4 d × 6 mg/kg	**1**	5/5	5/5
	**2**	0/5	0/5
4 d × 4 mg/kg	**1**	5/5	4/5
	**2**	0/5	0/5
4 d × 2 mg/kg	**1**	3/5	1/5
	**2**	0/5	0/5

a Mice were judged cured if parasitemia was undetectable at day 30. Results for these and four additional dosing regimens are provided in the Supporting Information.

The superior efficacy of **1** as compared to its regioisomer **2** seems unlikely to be attributable to its ∼2-fold superior *in vitro* potency alone. More likely, the special pharmacokinetic properties^2^ of **1** lead to superior *in vivo* drug exposure and efficacy against erythrocytic parasites. We further speculate that the poor aqueous solubility of **1** may paradoxically contribute to its superior efficacy *in vivo*; insoluble drug substance in the gastro-intestinal tract may be very slowly absorbed, conferring a drug depot effect, and producing a prolonged plasma exposure profile that drives single-dose efficacy. However, the QD × 4 day study arms reveal that even when administered over several days to mimic slow absorption, the efficacy of **2** does not approach that of **1**. The exposure profile of **2** in mice is no doubt inferior to **1**, despite its superior *in vitro* stability in human microsomes. In any event, new drug candidates from the trioxolane class should certainly possess improved solubility and rates of dissolution when compared to **1**. In this light, the results of this preliminary study are highly encouraging, and suggest that a desymmetrized artefenomel pharmacophore could provide a path toward differentiated physiochemical properties without abrogating the intrinsic antiplasmodial and antimalarial properties of this privileged scaffold.

Here we described a stereocontrolled synthesis of RLA-3107 (**2**), a regioisomer of the clinically studied drug candidate artefenomel. We confirmed the predictions of conformational analysis that *trans* relative stereochemistry of 3″-aryl substituted trioxolanes should afford similar rates of reactivity with ferrous iron and comparable antiplasmodial effects *in vitro*. The *in vitro* and *in vivo* studies described here validate and credential the 3″-aryl substituted trioxolane scaffold as an auspicious starting point for the discovery of next-generation endoperoxide development candidates. Efforts along these lines are underway and will be reported in due course.

## Experimental Methods

### Materials

All chemical reagents were obtained commercially and used without further purification, unless otherwise stated. Anhydrous solvents were purchased from Sigma-Aldrich and used without further purification. Solvents used for flash column chromatography and reaction workup procedures were purchased from either Sigma-Aldrich or Fisher Scientific. Column chromatography was performed on Silicycle Sili-prep cartridges using a Biotage Isolera Four automated flash chromatography system.

### Instrumentation

NMR spectra were recorded on either a Varian INOVA 400 MHz spectrometer (with 5 mm Quad-Nuclear Z-Grad Probe), or a Bruker AvanceIII HD 400 MHz (with 5 mm BBFO Z-gradient Smart Probe), calibrated to CH(D)Cl_3_ as an internal reference (7.26 and 77.00 ppm for ^1^H and ^13^C NMR spectra, respectively). Data for ^1^H NMR spectra are reported in terms of chemical shift (δ, ppm), multiplicity, coupling constant (Hz), and integration. Data for ^13^C NMR spectra are reported in terms of chemical shift (δ, ppm), with multiplicity and coupling constants in the case of C–F coupling. The following abbreviations are used to denote the multiplicities; s = singlet, d = doublet, t = triplet, q = quartet, m = multiplet, br = broad, app = apparent, or combinations of these. LC-MS and compound purity were determined using Waters Micromass ZQ 4000, equipped with a Waters 2795 Separation Module, Waters 2996 Photodiode Array Detector, and a Waters 2424 ELSD. Separations were carried out with an XBridge BEH C18, 3.5 μm, 4.6 × 20 mm column, at ambient temperature (unregulated) using a mobile phase of water–methanol containing a constant 0.10% formic acid.

#### Synthetic Procedures

##### 3-(4-Acetoxyphenyl)cyclohexan-1-one (**3**)

To an oven-dried round-bottom flask containing a Teflon-coated magnetic stir bar under an Ar(g) atmosphere was added palladium(II) acetylacetonate (0.156 g, 0.506 mmol, 0.05 equiv), 1,2-bis(diphenylphosphino)benzene (0.231 g, 0.506 mmol, 0.05 equiv), copper(II) tetrafluoroborate hydrate (0.484 g, 2.025 mmol, 0.2 equiv), and 4-acetoxyphenylboronic acid (2.789 g, 15.19 mmol, 1.5 equiv). To the mixture of solid materials was added anhydrous dimethoxyethane (60 mL). At this point, the solution appeared dark brown in color. To the stirring solution was then added 2-cyclohexen-1-one (1 mL, 10.12 mmol, 1.0 equiv) via syringe at room temperature. Within 5 min the solution had turned lime green in color. The solution was allowed to stir at room temperature for 16 h. Based on LCMS and TLC analysis, the reaction was judged incomplete and the following was added: palladium(II) acetylacetonate (0.078 g, 0.26 mmol, 0.025 equiv), 1,2-bis(diphenylphosphino)benzene (0.115 g, 0.26 mmol, 0.025 equiv), copper(II) tetrafluoroborate hydrate (0.242 g, 1.02 mmol, 0.1 equiv), and 4-acetoxyphenylboronic acid (1.39 g, 7.75 mmol, 0.75 equiv). The reaction mixture was stirred at room temperature for an additional 24 h, at which point reaction was judged complete. The mixture was then concentrated under reduced pressure to yield a dark green oil. To this oil was added EtOAc (100 mL) followed by H_2_O (50 mL). The layers were separated and the organic phase was washed with additional H_2_O (50 mL). The combined organic layer was then filtered through a pad of Celite, rinsing with EtOAc (50 mL × 4). The aqueous layer was further extracted with EtOAc, and the resulting organic filtered through the same pad of Celite, rinsing with EtOAc (50 mL × 2). The filtrate was concentrated under reduced pressure to yield a yellow oil that was purified by flash column chromatography (220 g silica gel cartridge, 0–20% EtOAc/hexanes) to yield **3** (2.133 g, 91%) as a foamy solid. ^1^H NMR (400 MHz, CDCl_3_) δ 7.22 (d, *J* = 8.5 Hz, 2H), 7.04 (d, *J* = 8.5 Hz, 2H), 3.08–2.93 (m, 1H), 2.64–2.55 (m, 1H), 2.55–2.42 (m, 2H), 2.42–2.32 (m, 1H), 2.29 (s, 3H), 2.19–2.03 (m, 2H), 1.90–1.70 (m, 2H); ^13^C NMR (100 MHz, CDCl_3_) δ 210.8, 169.6, 149.2, 141.8, 127.5, 121.7, 48.9, 44.1, 41.1, 32.7, 25.4, 21.1; MS (ESI) calculated for C_12_H_15_O_2_ [M + H]^+^*m*/*z* 191.11, found 190.90. This mass corresponds to loss of the acetate, followed by protonation of the phenoxide to yield the phenol.

##### 4-(Dispiro[Adamantane-2,3′-[1,2,4]trioxolane-5′,1’’-cyclohexan]-3′’-yl)phenyl Acetate (**4**)

To a solution of adamantan-2-one *O*-methyl oxime (3.23 g, 18.0 mmol, 2.0 equiv) in 100 mL carbon tetrachloride was added ketone **3** (2.09 g, 9.0 mmol, 1.0 equiv) as a solution in carbon tetrachloride (80 mL). This solution was then cooled to 0 °C and subsequently sparged with O_2_ for 10 min. The reaction was kept at 0 °C while ozone was then bubbled (2 L/min, 35% power). After stirring for 35 min, the reaction was deemed to be incomplete based on LCMS analysis and additional oxime (0.806 g, 4.50 mmol, 0.5 equiv) was added in a single portion to the reaction mixture. Ozone was bubbled through the reaction for another 60 min, at which point additional oxime (0.806 g, 4.50 mmol, 0.5 equiv) was added in a single portion to the reaction. The ozone power was increased to 40% and ozone addition was continued for another 80 min until the reaction was judged complete by TLC and HPLC analysis. The reaction mixture was then purged of ozone by bubbling with O_2_ for 10 min, followed argon gas for 10 min to remove dissolved oxygen. The solution was then concentrated under reduced pressure to provide a viscous oil. The residue was purified via flash column chromatography (220 g silica gel cartridge, 0–10% EtOAc/Hexanes) to yield **4** (3.60 g, 100%) as a thick colorless oil that solidified to a white solid upon standing in the refrigerator. The diastereoselectivity of reaction was determined by ^1^H NMR to be 8.1:1 in favor of the *trans* diastereomer. ^1^H NMR (400 MHz, CDCl_3_) δ 7.24–7.18 (m, 2H), 7.05–6.98 (m, 2H), 2.95 (tt, *J* = 12.7, 3.1 Hz, 1H, minor diastereomer), 2.81 (tt, *J* = 12.8, 3.3 Hz, 1H), 2.29 (s, 3H), 2.18–2.09 (m, 1H), 2.04–1.55 (m, 20H), 1.44–1.30 (m, 1H); ^13^C NMR (100 MHz, CDCl_3_) δ 169.6, 148.9, 143.3 (minor diastereomer), 143.1, 127.7, 121.4, 111.8 (minor diastereomer), 111.3, 108.9, 46.9 (minor diastereomer), 42.1, 41.6 (minor diastereomer), 41.3, 41.0 (minor diastereomer), 39.2 (minor diastereomer), 36.7, 36.4, 36.3 (minor diastereomer), 35.0 (minor diastereomer), 34.9 (minor diastereomer), 34.8, 34.7, 34.1, 34.0 (minor diastereomer), 33.5 (minor diastereomer), 32.7, 27.4 (minor diastereomer), 26.9 (minor diastereomer), 26.8, 26.4, 23.5, 21.1; MS (ESI) calculated for C_24_H_30_O_5_Na [M + Na]^+^*m*/*z* 421.20, found 420.95.

##### 4-(Dispiro[Adamantane-2,3′-[1,2,4]trioxolane-5′,1’’-cyclohexan]-3′’-yl)phenol (**5**)

To a solution of trioxolane **4** (3.53 g, 8.86 mmol, 1.0 equiv) in anhydrous THF (35 mL) and MeOH (70 mL) was added 15% aqueous KOH (15 mL, 40.10 mmol, 4.53 equiv). The solution was then placed in an oil bath that had been preheated to 50 °C, and was allowed to stir at this temperature for 2.5 h. During this time, the solution turned from a clear colorless solution to one that was dark brown in color. Upon determination of reaction completion by LCMS and TLC, the solution was removed from the oil bath and allowed to cool to room temperature. The mixture was then concentrated under reduced pressure to remove all volatile organic materials, which produced a viscous brown solution. To this was then added H_2_O (30 mL), which resulted in the formation of a yellow precipitate. Glacial acetic acid was then added to this solution until pH was around 4. This resulted in the formation of large tan colored solids to form. Some of the precipitate sank to the bottom of the flask, whereas some was observed to float on top of the solution as well. As a result, the aqueous solution was decanted by pouring the water onto a fritted funnel. The solid material collected on the frit was then dissolved with EtOAc and this solution was then added to the remaining solid in the original flask from which the aqueous solution was decanted out of. To this flask was added additional EtOAc (100 mL) to fully dissolve all of the solid material. This solution was then washed with DI H_2_O (40 mL × 2), Brine (40 mL), dried over anhydrous Na_2_SO_4_, filtered, and concentrated under reduced pressure to yield a thick brown oil. The residue was then purified through flash column chromatography (120 g HP silica gel cartridge, 0–20% EtOAc/Hexanes, product eluted during 7–10% EtOAc/Hex) to yield **4** (3.08 g, 98%) as a foamy solid that was determined to be a 15.3:1 mixture in favor of the *trans* diastereomer. ^1^H NMR (400 MHz, CDCl_3_) δ 7.12–7.02 (m, 2H), 6.81–6.72 (m, 2H), 4.95 (br s, 1H), 2.89 (tt, *J* = 12.6, 3.3 Hz, 1H, minor diastereomer), 2.74 (tt, *J* = 12.8, 3.3 Hz, 1H), 2.15–2.08 (m, 1H), 2.05–1.64 (m, 19H), 1.64–1.55 (m, 1H), 1.40–1.28 (m, 1H); ^13^C NMR (100 MHz, CDCl_3_) δ 153.9, 138.0, 127.8, 115.2, 111.4, 109.1; 42.3, 41.8 (minor diastereomer), 41.0, 40.7 (minor diastereomer), 36.8, 36.4, 35.0 (minor diastereomer), 34.9 (minor diastereomer), 34.8, 34.7, 34.2, 33.0, 26.8, 26.5, 23.5, 21.1 (minor diastereomer); MS (ESI) calculated for C_22_H_27_O_4_ [M – H]^−^*m*/*z* 355.19, found 355.15.

##### 4-(2-(4-(Dispiro[Adamantane-2,3′-[1,2,4]trioxolane-5′,1’’-cyclohexan]-3′’-yl)phenoxy)ethyl)morpholine (**2**)

To a solution of phenol **5** (100 mg, 0.28 mmol, 1.0 equiv) in dry CH_3_CN (5 mL) was added tetrabutylammonium hydrogen sulfate (19.1 mg, 0.056 mmol, 0.2 equiv) and powdered NaOH (44.9 mg, 1.12 mmol, 4.0 equiv). This mixture was then allowed to stir at room temperature for 30 min, at which point 4-(2-chloroethyl)morpholine hydrochloride (104.4 mg, 0.56 mmol, 2.0 equiv) was added to the solution. The mixture was then placed in an oil bath preheated to 55 °C, and was allowed to stir at this temperature for 14.5 h after which the reaction was judged complete. After cooling, the mixture was diluted with EtOAc (20 mL) and H_2_O (10 mL), which served to dissolve all of the inorganic solids present. Following separation of the layers, the aqueous layer was extracted with EtOAc (20 mL) which resulted in the formation of an emulsion, to which brine (10 mL) was added to aid in separation of the organic phase. The combined organic layers were then washed with Brine (10 mL), dried over anhydrous Na_2_SO_4_, filtered, and concentrated under reduced pressure. The residue was then purified via flash column chromatography (25 g silica gel cartridge, 0–100% EtOAc/Hexanes) to yield **2** (82 mg, 62%) as a clear colorless oil that was a single diastereomer by ^1^H NMR analysis. ^1^H NMR (400 MHz, CDCl_3_) δ 7.11 (d, *J* = 8.6 Hz, 2H), 6.84 (d, *J* = 8.6 Hz, 2H), 4.09 (t, *J* = 5.7 Hz, 2H), 3.74 (t, *J* = 4.7 Hz, 4H), 2.79 (t, *J* = 5.7 Hz, 2H), 2.79–2.68 (m, 1H), 2.58 (t, *J* = 4.5 Hz, 4H), 2.15–2.06 (m, 1H), 2.04–1.87 (m, 7H), 1.87–1.54 (m, 13H), 1.40–1.24 (m, 1H); ^13^C NMR (100 MHz, CDCl_3_) δ 157.0, 138.1, 127.6, 114.5, 111.3, 109.0, 66.8, 65.7, 57.6, 54.0, 42.3, 41.0, 36.7, 36.4, 34.8, 34.7, 34.1, 32.9, 26.8, 26.4, 23.5; MS (ESI) calculated for C_28_H_40_NO_5_ [M + H]^+^*m*/*z* 470.29, found 470.09.

##### *Plasmodium falciparum* EC_50_ Determination

The growth inhibition assay for *P. falciparum* was conducted as described previously^18^ with minor modifications. Briefly, *P. falciparum* strain W2 synchronized ring-stage parasites were cultured in human red blood cells in 96-well flat bottom culture plates at 37 °C, adjusted to 1% parasitemia and 2% hematocrit under an atmosphere of 3% O_2_, 5% CO_2_, 91% N_2_ in a final volume of 0.1 mL per well in RPMI-1640 media supplemented with 0.5% Albumax, 2 mM l-glutamine and 100 mM hypoxanthine in the presence of various concentrations of inhibitors. Test compounds were serially diluted 1:3 in the range 10,000–4.6 nM (or 1,000–0.006 nM for more potent analogs), with a maximum DMSO concentration of 0.1%. Following 48 h of incubation, the cells were fixed by adding 0.1 mL of 2% formaldehyde in phosphate buffered saline, pH = 7.4 (PBS). Parasite growth was evaluated by flow cytometry on a FACsort (Becton Dickinson) equipped with AMS-1 loader (Cytek Development) after staining with 1 nM of the DNA dye YOYO-1 (Molecular Probes) in 100 mM NH_4_Cl, 0.1% Triton x-100 in 0.8% NaCl. Parasitemias were determined from dot plots (forward scatter vs fluorescence) using CELLQUEST software (Becton Dickinson). EC_50_ values for growth inhibition were determined from plots of percentage control parasitemia over inhibitor concentration using GraphPad Prism software.

##### *P. berghei* Mouse Malaria Model

Female Swiss Webster mice (∼20 g body weight) were infected intraperitoneally with 10^6^*P. berghei*-infected erythrocytes collected from a previously infected mouse. Beginning 1 h after inoculation the mice were treated once daily by oral gavage for 1–4 days with 100 μL of solution of test compound formulated in 10% DMSO, 40% of a 20% 2-hydroxypropyl-beta-cyclodextrin solution in water, and 50% PEG400. There were five mice in each test arm. Infections were monitored by daily microscopic evaluation of Giemsa-stained blood smears starting on day seven. Parasitemia was determined by counting the number of infected erythrocytes per 1000 erythrocytes. Body weight was measured over the course of the treatment. Mice were euthanized when parasitemia exceeded 50% or when weight loss of more than 15% occurred. Parasitemia, animal survival, and morbidity were closely monitored for 30 days postinfection, when experiments were terminated.

##### Animal Welfare

No alternative to the use of laboratory animals is available for in vivo efficacy assessments. Animals were housed and fed according to NIH and USDA regulations in the Animal Care Facility at San Francisco General Hospital. Trained animal care technicians provide routine care, and veterinary staff are readily available. Euthanasia was performed when malarial parasitemias top 50%, a level that does not appear to be accompanied by distress, but predicts progression to lethal disease. Euthanasia was accomplished with CO_2_ followed by cervical dislocation. These methods are in accord with the recommendations of the Panel on Euthanasia of the American Veterinary Medical Association. Our studies are approved by the UCSF Committee on Animal Research.

## Supporting Information Available

Table describing the full results of in vivo studies with **1** and **2** (PDF)
