# Supplementary material for: Artefenomel Regioisomer RLA-3107 Is a Promising Lead for the Discovery of Next-Generation Endoperoxide Antimalarials
Source: ACS Med Chem Lett. 2023 Apr 4;14(4):493–8. doi: 10.1021/acsmedchemlett.3c00039 (PMC10108391; doi:10.1021/acsmedchemlett.3c00039)
Supplement: Supplementary file 1 — ml3c00039_si_001.pdf [file ml3c00039_si_001.pdf]

Supporting information for

Artefenomel regioisomer RLA-3107 is a promising lead for the discovery of next-generation endoperoxide antimalarials

Brian R. Blank,[†] Jiri Gut,[§] Philip J. Rosenthal, and Adam R. Renslo[†,*]

†Department of Pharmaceutical Chemistry, University of California, San Francisco, 600 16th Street, San Francisco, CA 94158, United States.

§Department of Medicine, San Francisco General Hospital, University of California, San Francisco, California 94143, United States.

Table of Contents

Table S1………………………………..S2

Table S1. In vivo efficacy of compounds **1** (artefenomel) and **2** (RLA-3107) across twelve in vivo study arms with distinct dosing schedules.

		percent survivors (n = 5)									
	**Days x PO Dose**	**7**	**8**	**9**	**10**	**11**	**13**	**16**	**21**	**24**	**30**
**Compound 2**	**1x80 mg/kg**	100	100	100	100	100	100	100	100	100	100
**Compound 2**	**1x40mg/kg**	100	100	100	100	60	20	20	20	20	20
**Compound 2**	**1x30mg/kg**	100	100	100	100	100	0	0	0	0	0
**Compound 2**	**1x20mg/kg**	100	100	100	100	60	0	0	0	0	0
**Compound 2**	**1x10mg/kg**	100	80	0	0	0	0	0	0	0	0
**Compound 2**	**1x4mg/kg**	0	0	0	0	0	0	0	0	0	0
**Compound 2**	**2x4mg/kg**	60	0	0	0	0	0	0	0	0	0
**Compound 2**	**3x4mg/kg**	80	0	0	0	0	0	0	0	0	0
**Compound 2**	**4x10mg/kg**	100	100	100	80	80	60	0	0	0	0
**Compound 2**	**4x6mg/kg**	80	40	0	0	0	0	0	0	0	0
**Compound 2**	**4x4mg/kg**	0	0	0	0	0	0	0	0	0	0
**Compound 2**	**4x2mg/kg**	0	0	0	0	0	0	0	0	0	0
**Compound 1**	**1x80mg/kg**	100	100	100	100	100	100	100	100	100	100
**Compound 1**	**1x40mg/kg**	100	100	100	100	100	100	100	100	100	100
**Compound 1**	**1x30mg/kg**	100	100	100	100	100	100	100	100	100	100
**Compound 1**	**1x20mg/kg**	100	100	100	100	100	100	100	100	100	100
**Compound 1**	**1x10mg/kg**	100	100	100	100	100	100	100	100	100	100
**Compound 1**	**1x4mg/kg**	100	100	100	100	40	20	20	20	20	20
**Compound 1**	**2x4mg/kg**	100	100	100	100	100	40	40	40	40	40
**Compound 1**	**3x4mg/kg**	100	100	100	100	100	100	60	60	60	60
**Compound 1**	**4x10mg/kg**	100	100	100	100	100	100	100	100	100	100
**Compound 1**	**4x6mg/kg**	100	100	100	100	100	100	100	100	100	100
**Compound 1**	**4x4mg/kg**	100	100	100	100	100	100	80	80	80	80
**Compound 1**	**4x2mg/kg**	100	100	100	60	60	20	20	20	20	20
**Vehicle**		20	0	0	0	0	0	0	0	0	0
**Untreated**		40	0	0	0	0	0	0	0	0	0
